# Total lesion glycolysis on FDG-PET/CT before salvage surgery predicts survival in laryngeal or pharyngeal cancer

**DOI:** 10.18632/oncotarget.24914

**Published:** 2018-04-10

**Authors:** Hidenori Suzuki, Tsuneo Tamaki, Masami Nishio, Yusuke Nakata, Nobuhiro Hanai, Daisuke Nishikawa, Yusuke Koide, Yasuhisa Hasegawa

**Affiliations:** ^1^ Department of Head and Neck Surgery, Aichi Cancer Center Hospital, Nagoya, Japan; ^2^ Department of East Nagoya Positron Emission Tomography Imaging Center, Nagoya, Japan; ^3^ Department of Radiology, Nagoya Positron Emission Tomography Imaging Center, Nagoya, Japan; ^4^ Department of Otorhinolaryngology, Shiga University of Medical Science, Otsu, Japan

**Keywords:** laryngeal and pharyngeal squamous cell carcinoma, 18F-FDG-PET/CT, total lesion glycolysis, overall survival, lung metastasis

## Abstract

We investigated whether 18F-fluorodeoxyglucose uptake parameters using positron emission tomography combined with computed tomography predicts several survival outcomes, including lung metastasis-free survival, in patients with laryngeal or pharyngeal cancer who underwent salvage surgery. The maximum standardized uptake value, metabolic tumor volume, and total lesion glycolysis were calculated as 18F-fluorodeoxyglucose uptake parameters in 51 patients with laryngeal or pharyngeal cancer before salvage surgery. In univariate analysis, the maximum standardized uptake value ≥ 22.8, metabolic tumor volume ≥ 2.4, and total lesion glycolysis ≥ 5.4 were significantly correlated with shorter overall survival. In multivariate analysis with adjustment for clinical stage, patients with total lesion glycolysis ≥ 5.4 exhibited significantly shorter overall survival. Furthermore, total lesion glycolysis ≥ 5.4 was significantly correlated with shorter disease-specific survival, distant metastasis-free survival, and lung metastasis-free survival in univariate analysis. In conclusion, total lesion glycolysis predicts the survival outcomes including lung metastasis in patients with laryngeal or pharyngeal cancer who underwent salvage surgery.

## INTRODUCTION

Salvage surgery is radical therapy for recurrence, residual and metachronous tumors after radiotherapy (RT) with or without chemotherapy in head and neck cancer, including laryngeal or pharyngeal squamous cell carcinoma (LPSCC) [1, 2]. The tumor-node-metastasis (TNM) staging system has difficulty estimating the survival outcomes for patients with the same TNM stage, such as those who underwent salvage surgery in LPSCC [3]. Several parameters for predicting the prognosis of patients undergoing salvage surgery in LPSCC have been evaluated via various approaches, such as imaging and pathological methods [1, 2].

The maximum standardized uptake value (SUVmax) is traditionally and semiquantitatively measured as18F-fluorodeoxyglocose (18F-FDG) uptake parameter for primary tumor in various types of cancer; it is assessed by pre- or posttreatment positron emission tomography combined with computed tomography (PET/CT) [4]. The metabolic tumor volume (MTV) as well as the total lesion glycolysis (TLG), which incorporates the MTV and mean standardized uptake value (SUVmean), have recently been measured as volumetric parameters on 18F-FDG-PET/CT using software programs [4]. Several pre-or posttreatment 18F-FDG-uptake parameters have been shown to be correlated with the overall survival (OS) in head and neck cancer, including various sites of primary tumor [4–10], and we have also reported in our study of 53 hypopharyngeal cancer that high TLG on pretreatment 18F-FDG-PET/CT at initial staging is correlated with a shorter OS and distant metastasis-free survival [5].

Previous studies in patients with LPSCC who underwent RT with or without chemotherapy have reported that posttreatment 18F-FDG-uptake parameters are significantly correlated with the OS [4, 6–9]. However, the association between 18F-FDG uptake parameters and the OS remains unclear in patients with LPSCC following salvage surgery. Furthermore, the association between 18F-FDG uptake parameters and several survival outcomes, including the lung metastasis-free survival, in LPSCC has not been thoroughly investigated.

In the present study, we investigated a possible correlation between 18F-FDG-uptake parameters assessed by pretreatment 18F-FDG-PET/CT and the OS in patients with LPSCC who underwent salvage surgery, and determined whether 18F-FDG-uptake parameters were correlated with several survival outcomes, including lung-metastasis free survival.

## RESULTS

### Clinicopathological parameters

Fifty-one patients were enrolled in this study. The mean duration ± standard deviation (SD) between pretreatment 18F-FDG-PET/CT and salvage surgery was 27.3 ± 14.3 days. The clinicopathological parameters of all patients are shown in Table 1.

**Table 1:** Clinicopathological parameters (*n* = 51)

Parameter
Age	Mean ± standard devaition	66.1 ± 7.71
Gender	Male/Female	48/3
Clinical T classification	Tis/T1/T2/T3/T4	1/17/17/7/9
Clinical N classification	N0/N1/N2/N3	46/4/1/0
Clinical stage	0/I/II/III/IV	1/16/15/9/10
Primary tumor site	Layrnx/Oroparynx/Hypopharynx	20/11/20
Diagnosis	Residual/Recurrence/Metachronous	5/44/2
History of surgery	Presence/Absence	19/32
History of chemotherapy	Presence/Absence	42/9
Reconstruction surgery	Presence/Absence	25/26
Pathological T classification	Tis/T1/T2/T3/T4	2/25/10/8/6
Pathological N classification	N0/N1/N2/N3	41/9/1/0
Pathological stage	0/I/II/III/IV	1/22/8/13/7
Positive surgical margin	Presence/Absence	10/41

### 18F-FDG-uptake parameters

**Figure 1:**
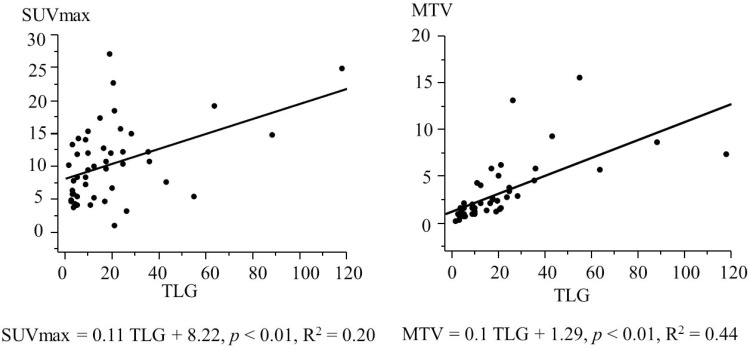
Simple regression analysis (SUVmax and TLG, MTV and TLG) in 47 patients with laryngeal or pharyngeal squamous cell carcinoma Abbreviations: SUVmax: maximum standardized uptake value; TLG: total lesion glycolysis; MTV: metabolic tumor volume.

The sensitivity of detecting the primary tumor site on 18F-FDG-PET/CT was 92.2% (47/51). False negatives, which were undetectable by 18F-FDG-PET/CT, occurred in two T1 laryngeal SCC patients, one T1 hypopharyngeal SCC patient, and one oropharyngeal SCC patient. Among the 47 patients with a primary tumor detected on 18F-FDG-PET/CT, the SUVmax, MTV, and TLG values (mean ± SD) of the primary tumor were 10.5 ± 5.8 g/ml, 3.2 ± 3.3 cm^3^, and 19.6 ± 22.6 g, respectively. TLG was significantly correlated with both the SUVmax (*p* < 0.01, R^2^ = 0.20) and MTV (*p* < 0.01, R^2^ = 0.44), as shown in Figure 1. In contrast, MTV was not significantly correlated with the SUVmax (SUVmax = –0.25 MTV + 11.2, *p* = 0.34, R^2^ = 0.02).

### Follow-up and survival outcomes

At the end of the study, the mean ± SD duration of follow-up from the date of 18F-FDG-PET/CT was 47.7 ± 23.6 months. The mean ± SD duration of follow-up for the 28 (54.9%, vs all) patients who are still alive, the 23 (45.1%, vs all) patients who died, the 20 (39.2%, vs all) patients who succumbed to LPSCC was 63.2 ± 16.4 months, 28.8 ± 16.0 months, 27.4 ± 14.6 months, respectively. Eleven (21.6%, vs all), 10 (19.6%, vs all), 15 (29.4%, vs all), and 11 (21.6%, vs all) developed local recurrence, regional recurrence, distant metastasis, and lung metastasis, respectively. The mean ± SD duration between 18F-FDG-PET/CT and local recurrence, regional recurrence, distant metastasis, and lung metastasis was 8.4 ± 7.3 months, 9.0 ± 5.0 months, 14.5 ± 11.6 months, and 11.7 ± 6.5 months, respectively.

### OS analysis

**Figure 2:**
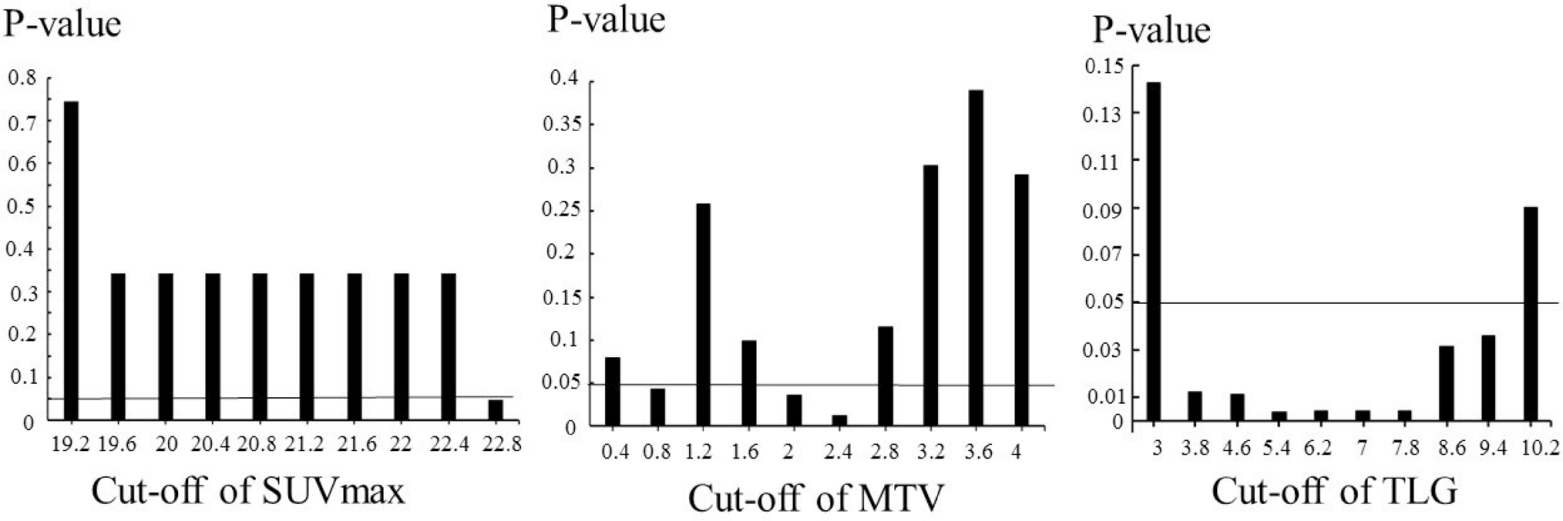
*P-*values of Wilcoxon’s test for the univariate overall survival analysis using different cut-off levels for 18F-fluorodeoxyglucose uptake parameters (SUVmax, MTV, TLG) of 51 patients with laryngeal or pharyngeal squamous cell carcinoma Abbreviations: SUVmax: maximum standardized uptake value; TLG: total lesion glycolysis; MTV: metabolic tumor volume.

**Figure 3:**
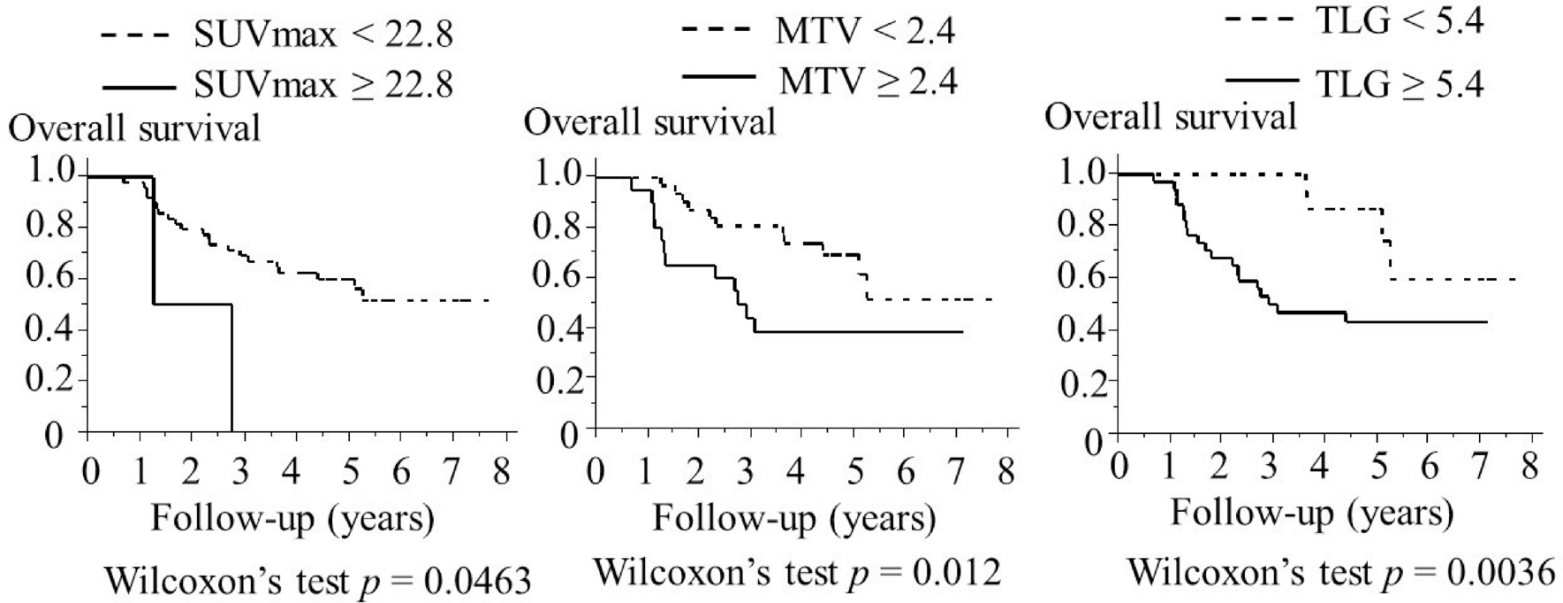
Association between 18F- fluorodeoxyglocose uptake parameters and the overall survival of 51 patients with laryngeal or pharyngeal squamous cell carcinoma (Kaplan–Meier method) SUVmax ≥ 15.2, MTV ≥ 2.4, and TLG ≥ 5.4 were associated with a significantly shorter overall survival. Wilcoxon’s test was used for the statistical analysis. Abbreviations: SUVmax: maximum standardized uptake value; TLG: total lesion glycolysis; MTV: metabolic tumor volume.

Various cut-off values for 18F-FDG uptake parameters (SUVmax, MTV, and TLG) were tested using Wilcoxon’s test in the univariate OS analysis. The cut-off values with the lowest *P-*values were SUVmax = 22.8, MTV = 2.4 and TLG = 5.4. The FDG uptake parameter cut-off values and *P* values are shown in Figure 2. SUVmax ≥ 22.8 (*p* = 0.0463), MTV ≥ 2.4 (*p* =0.012), and TLG ≥ 5.4 (*p* = 0.0036) were significantly effective in differentiating the shorter OS group from the longer OS group. When cut off values of TLG were from 6.2 to 7.8, all of the *p-*values was 0.0042. The Kaplan–Meier curves from the univariate OS analysis are shown in Figure 3. SUVmax ≥ 22.8 (*p* = 0.0249), MTV ≥ 2.4 (*p* = 0.0492), TLG ≥ 5.4 (*p* = 0.0178) were significantly divided the shorter OS group from the longer OS group by using log-rank test. In univariate OS analysis of the Cox proportional hazards model, SUVmax ≥ 22.8 was not significantly associated with a shorter OS than SUVmax < 22.8 (hazards ratio = 4.69, 95% confidence interval 0.73–16.9, *p* = 0.09), MTV ≥ 2.4 was not significantly correlated with a shorter OS than MTV < 2.4 (hazards ratio = 2.24, 95% confidence interval 0.98–5.19, *p* = 0.06), and TLG ≥ 5.4 was significantly associated with a shorter OS than TLG < 5.4 (hazards ratio = 3.42, 95% confidence interval 1.28–11.8, *p* = 0.01).

### Clinicopathological and 18F-FDG uptake parameters

The relationships between the clinicopathological parameters and 18F-FDG uptake parameters are shown in Table 2. There were no significant relationships between the SUVmax (<22.8/≥22.8) and clinicopathological parameters. An MTV ≥ 2.4 was significantly correlated with both a clinical T3-4 classification (*p* < 0.01) and clinical III-IV stage (*p* < 0.01). TLG ≥ 5.4 was significantly correlated with a clinical T3-4 classification (*p* < 0.01), clinical III-IV stage (*p* < 0.01), pathological T3-4 classification (*p* < 0.01), and pathological III-IV stage (*p* < 0.01).

**Table 2:** Relationsships between the clinicopathological parameters and the FDG uptake parameters by the 2 × 2 test

Parameter		SUVmax		MTV		TLG	
		≥22.8 (*n* = 2)	<22.8 (*n* = 49)	≥2.4 (*n* = 20)	<2.4 (*n* = 31)	≥5.4 (*n* = 34)	<5.4 (*n* = 17)
Age	≥66/<66	1/1	23/26	12/8	12/19	17/17	7/10
	*p-*value		1.00		0.16		0.77
Gender	Male/Female	2/0	46/3	20/0	28/3	32/2	16/1
	*p-*value		1.00		0.27		1.00
Clinical T	Tis-T2/T3-T4	0/2	35/14	9/11	26/5	18/16	17/0
classification	*p-*value		0.09		<0.01		<0.01
Clinical N classification	N0/N1-N2 *p-*value	2/0	44/5	17/3	29/2	30/4	16/1
			1.00		0.37		0.65
Clinical stage	0-II/III-IV *p-*value	0/2	32/17 0.13	8/12	24/7 <0.01	16/18	16/1 <0.01
Primary tumor site	Larynx/Pharynx *p-*value	1/1	19/30 1.00	8/12	12/19 1.00	12/22	8/9 0.55
Diagnosis	Residual/Others *p-*value	1/1	4/45 0.19	2/18	3/28 1.00	4/30	1/16 0.65
History of surgery	Presence/Absence *p-*value	1/1	18/31 1.00	7/13	12/19 1.00	13/21	6/11 1.00
History of chemotherapy	Presence/Absence *p-*value	2/0	40/9 1.00	16/4	26/5 0.72	29/5	13/4 0.46
Reconstruction surgery	Presence/Absence *p-*value	2/0	23/26 0.24	12/8	13/18 0.26	20/14	5/12 0.07
Pathological T classification	Tis-T2/T3-T4 *p-*value	0/2	37/12 0.07	11/9	26/5 0.05	20/14	17/0 <0.01
Pathological N classification	N0/N1-N2 *p-*value	2/0	39/10 1.00	14/6	27/4 0.16	25/9	16/1 0.14
Pathological stage	0-II/III-IV *p-*value	0/2	31/18 0.15	9/11	22/9 0.08	15/19	16/1 <0.01
Positive surgical margin	Presence/Absence *p-*value	0/2	9/40 1.00	2/18	7/24 0.45	5/29	4/13 0.46

Abbreviations: FDG: fluorodeoxyglucose; SUVmax: the maximum standardized uptake value; MTV: metabolic tumor volume; TLG: total lesion glycolysis.

### Multivariate OS analysis

We performed a multivariate OS analysis adjusted for clinical stage (III-IV/I-II) to determine the factors associated with the OS. TLG ≥ 5.4 was found to be associated with a shorter OS than TLG < 5.4 (hazards ratio = 3.14, 95% confidence interval 1.04–11.57, *p* < 0.05). A multivariate analysis of the OS is shown in Table 3.

**Table 3:** A multivariate overall survival analysis by the Cox proportional hazards model

Parameter		Hazards ratio	95% confidence interval	*P-*value
Model 1-SUVmax				
SUVmax	(≥22.8/<22.8)	3.40	0.51–13.86	0.18
Clinical stage	(III-IV/0-II)	1.66	0.68–3.93	0.26
Model 2-MTV				
MTV	(≥2.4/<2.4)	1.92	0.75–4.88	0.17
Clinical stage	(III-IV/0-II)	1.42	0.56–3.58	0.46
Model 3-TLG				
TLG	(≥5.4/<5.4)	3.14	1.04–11.57	<0.05
Clinical stage	(III-IV/0-II)	1.19	0.49–2.96	0.71

Abbreviations: SUVmax: the maximum standardized uptake value; MTV: metabolic tumor volume; TLG: total lesion glycolysis.

### Survival outcomes between TLG ≥ 5.4 and TLG < 5.4

**Figure 4:**
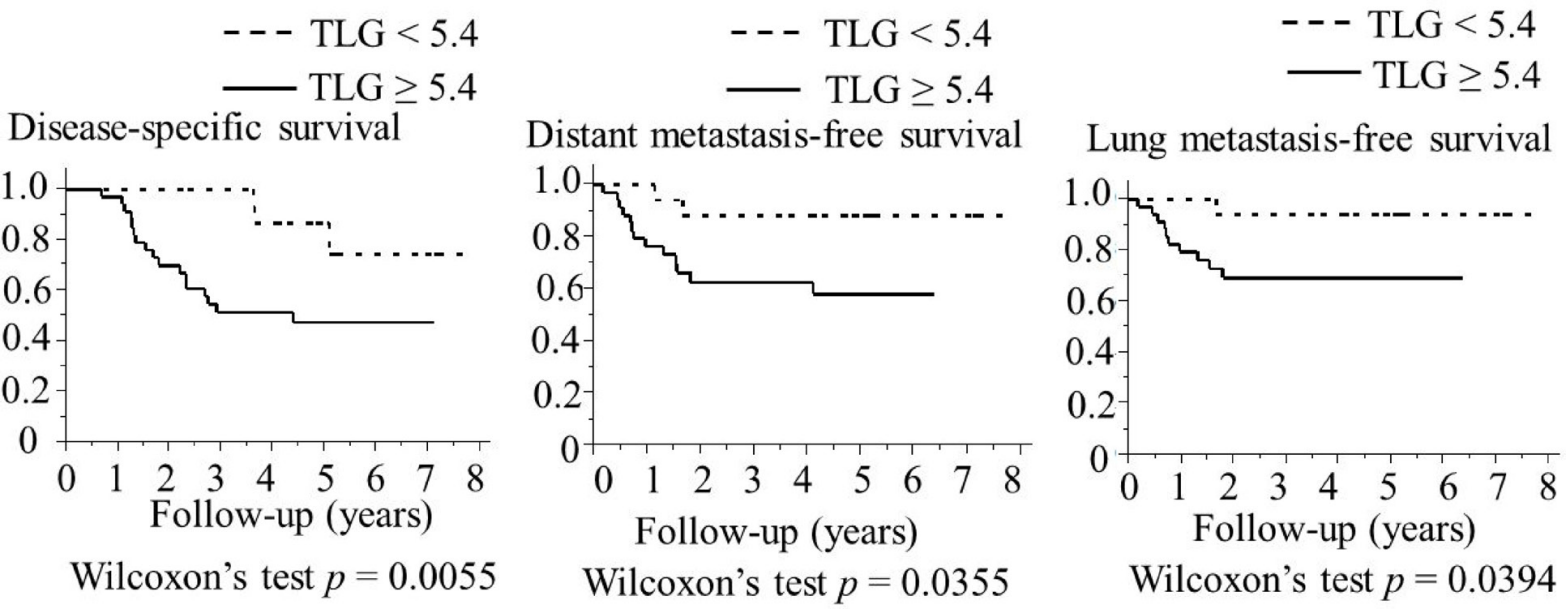
Association between TLG and the survival of 51 patients with laryngeal or pharyngeal squamous cell carcinoma (Kaplan–Meier method) TLG ≥ 5.4 was found to be associated with a significantly lower disease-specific survival, distant metastasis-free survival, and lung metastasis-free survival by Wilcoxon’s test. Abbreviations: TLG: total lesion glycolysis.

The patients with TLG ≥ 5.4 had a significantly shorter disease-specific survival (*p* < 0.0055), distant metastasis-free survival (*p* < 0.0355), and lung metastasis-free survival (*p* < 0.0394) than those with TLG < 5.4. However, no significant difference was noted in the locoregional recurrence-free survival (*p* = 0.16). The Kaplan–Meier curves for the disease- specific survival, distant metastasis-free survival, and lung metastasis-free survival are shown in Figure 4.

## DISCUSSION

In this study, we showed for the first time that TLG ≥ 5.4 was significantly correlated with a shorter OS in patients with LPSCC who underwent salvage surgery in univariate and multivariate analyses, and TLG ≥ 5.4 was significantly correlated with a shorter disease- specific survival, distant metastasis-free survival, and lung metastasis-free survival in a univariate analysis.

The SUVmax is a single-voxel representation of the maximum 18F-FDG uptake, the MTV is the tumor volume calculated by the distribution of metabolic activity, and TLG theoretically represents the total activity of all metabolically active cancer cells [4]. We previously reported in a simple regression analysis of 50 primary tumors with hypopharyngeal SCC assessed by pretreatment 18F-FDG-PET/CT at initial staging that TLG closely correlated with both the SUVmax (*p* < 0.01, R^2^ = 0.15) and MTV (*p* < 0.84, R^2^ = 0.84) [5]. Our present findings showed that TLG is significantly correlated with both the SUVmax and MTV, findings which agree with those in our previous study [5].

Although several studies have reported no significant association between the 18F-FDG uptake parameters and the OS in a single institution study [10, 11], significant correlations have been noted in meta-analyses and reviews for the prediction of 18F-FDG uptake parameters and OS in head and neck cancer [4, 6–8]. For example, Pak K *et al.* reported in a meta-analysis of 13 studies including 1180 patients that the OS was significantly correlated with the SUVmax, MTV, and TLG which were assessed as pretreatment 18F-FDG uptake parameters [6], and Sheilbahaei S *et al.* reported in a meta-analysis of 21 studies that the OS was significantly correlated with pre- or posttreatment 18F-FDG uptake parameters [7]. In addition, several authors have shown in reviews that high 18F-FDG uptake parameters were associated with an increased risk of death [4, 8]. Our present findings showed a significant relationship between high FDG-uptake parameters (SUVmax, MTV, and TLG) and a shorter OS, findings which are in agreement with those in previous reviews and meta-analyses [4, 6–8].

Regarding the correlation between the prognosis and 18F-FDG uptake parameters in patients with head and neck cancer who underwent salvage surgery, Kunkel M *et al.* reported in 41 patients with oral SCC that tumors detected by PET before salvage surgery were associated with a significantly worse disease-specific survival than tumors that were not detected by PET, but there was no mention of any association with 18F-FDG uptake parameters, such as TLG, before salvage surgery [9].

As the reason for clinical stage and not T-stage in multivariate analyses of the present study, we thought that clinical stage is suitable to adjust multivariate analyses due to comprehensive stage based on both T and N stages.

Regarding the close correlation between the prediction of distant metastasis and TLG in LPSCC, Lim R *et al* reported that TLG on pretreatment PET/CT at initial staging was associated with distant metastasis in cases of oropharyngeal SCC [12], and we also reported a significant correlation in patients newly diagnosed with hypopharyngeal SCC between high TLG and a shorter distant metastasis-free survival [5]. Our present findings demonstrated a significant relationship between high TLG and a shorter distant metastasis- free survival, findings which are in agreement with those in previous studies [5, 12].

Lung metastasis is the most common site of distant metastasis in LPSCC [13–15]. Because the association between TLG and lung metastasis has not been investigated in patients with LPSCC who underwent salvage surgery, we needed to investigate survival outcomes including lung metastasis-free survival in the present study. Based on the significant correlation between high TLG and a shorter lung metastasis-free survival in the present study, TLG on pretreatment PET/CT at salvage surgery in LPSCC seems to be a prognostic parameter for identifying patients at high risk group of developing lung metastasis.

Pak K, *et al.* reported in a systemic review and meta-analysis for prognostic value of FDG-uptake parameters that cut-off values are determined mostly by the lowest *p-*value, receiver-operating characteristic, or a median value, and that the lowest *p-*value method has widely been used [6]. Moreover, we also had used the lowest *p-*value method for the determination of the cut-off values in previous study for prognostic value of FDG-uptake parameters [5].

The limitations of the present study include retrospective design and the relatively small number of patients. Another limitation of the present study contains relatively heterogeneous cohort in regards to treatment, which will have an impact on survival outcomes. Prospective research with large sample sizes will allow for more statistically significant outcomes.

In conclusion, the present study demonstrated that high TLG is significantly correlated with a lower OS, disease-specific survival, distant metastasis-free survival, and lung metastasis-free survival in patients with LPSCC who underwent salvage surgery. The results of the present study suggest that TLG on pretreatment 18F-FDG-PET/CT at salvage surgery is a prognostic parameter in individuals with LPSCC.

## MATERIALS AND METHODS

### Study design and patients

Between 2008 May and 2012 July, 53 patients with a history of RT for the surgery area who had primary tumor with squamous cell carcinoma in the pharynx or larynx or cervical esophagus by a pathological examination and had been treated by salvage surgery at Aichi Cancer Center Hospital underwent pretreatment 18F-FDG-PET/CT at East Nagoya Positron Emission Tomography Imaging Center. Of these 53 patients, 2 patients with cervical esophageal cancer were excluded. Therefore, 51 patients were included in this study. This retrospective study was approved by the institutional review board, and informed consent for the treatments and inspections was given by all of the patients.

### Past history of treatment and salvage surgery

All patients were treated by RT with or without chemotherapy at a total dose 64.7 ± 11.8 Gy for the pharynx or larynx. Forty-three patients were treated by definitive RT with or without chemotherapy, and 8 patients were treated by postoperative RT with or without chemotherapy. Nineteen patients had a history of surgery for the pharynx or larynx. Clinical staging was determined from a routine physical examination, nasopharyngoscopy, enchanced cervical computed tomography or magnetic response imaging, a pathological examination of biopsy, and 18F-FDG-PET/CT. We performed both preoperative staging (T and N classification, stage) based on the International Union Against Cancer, seventh edition, and salvage surgery was conducted for 44 recurrent, 5 residual, 2 metachronous tumors. Twenty-five patients underwent reconstruction surgery after primary tumor resection with neck dissection, and 26 patients underwent no reconstruction after primary tumor resection with or without neck dissection. At the outpatient clinic, we made an effort to diagnose the patients with early locoregional recurrence and performed salvage therapy.

## 18F-FDG-PET/CT

All patients were scanned with Biograph True Point PET/CT/40 with True V (Siemens Health Medical Solutions Inc., Malvern, PA, USA), and low-dose (4.3 mSv) CT images for the RANDO Phantom (Alderson Research Laboratories Inc., Long Island, NY, USA) were used for attenuation correction of the PET data, in accordance to the methods described previously [5]. The mean ± SD blood sugar level at the date of staging was 109.5 ± 29.0 mg/dL. Patients were not eaten for 6 h before intravenous drip of 18F-FDG, and images were acquired 90 min after intravenous infusion of the tracer. A focus was deemed positive if the activity was significantly above the anticipated background level, and the boundaries were mechanically drawn to contain the primary tumor within the pharynx or larynx by three-dimensional (3D) 18F-FDG-PET/CT images. The 3D 18F-FDG-PET/CT images were created from PET/CT findings in SUV mode for a semiquantitative evaluation using the Advantage Workstation 4.6 software program PET VCAR (GE Healthcare, Chalfont, UK), and the FDG-uptake was defined as the SUV, which was determined using the following formula: SUV = Tissue concentration (Bq/g)/[injection dose (Bq)/body weight (g)]. The SUVmax of the primary tumor was automatically calculated by a volumetric region of interest on several consequent 3D images. According to our previous report,^5^ both the MTV and SUV mean from the volume of interest, which contain primary tumor only, were calculated by adopting 45% threshold fraction of the SUVmax, and TLG was determined using the following formula: TLG = MTV × SUVmean.

### Statistical analysis

Statistical analyses were performed in the JMP software package (version 9; SAS; Cary, NC, USA). Among the 47 patients with a primary tumor detected on 18F-FDG-PET/CT, the correlation between 18F-FDG-uptake parameters (SUVmax, MTV, TLG) were analyzed by a simple regression analysis.

In all cases, the OS time, which was calculated by the Kaplan–Meier method, was defined as the period from 18F-FDG-PET/CT to death or last contact. In accordance with the previous method, we determined the cut-off values for various FDG-uptake parameters (SUVmax, MTV, TLG) in a univariate OS analysis, which was carried out using the Wilcoxon test.^3^ Although there were some censor events in the present study, we selected Wilcoxon’s test which regards deaths in the earlier periods as important. We investigated the association between OS and cut-off values of FDG-uptake parameters (SUVmax = 22.8, MTV = 2.5 and TLG = 22.8) by log-rank test. A univariate OS analysis of a Cox proportional hazards model was used to assess numbers at risk for the Kaplan–Meier curves and confidence intervals for cut-off values of FDG-uptake parameters (SUVmax = 22.8, MTV = 2.4 and TLG = 5.4). In the univariate OS analysis, all patients were separated into 2 groups based on the SUVmax (SUVmax ≥ 22.8; SUVmax < 22.8), MTV (MTV ≥ 2.4; MTV < 2.4), TLG (TLG ≥ 5.4; TLG < 5.4). The relationships between the two groups of each FDG-uptake parameter (SUVmax, MTV, TLG) with the clinicopathological parameters (age, gender, clinical T and N classification, clinical stage, primary tumor site, diagnosis, history of surgery and chemotherapy, reconstruction surgery, pathological T and N classification, pathological stage, positive surgical margin) were compared using a 2 × 2 test. A multivariate OS analysis with adjustment for the clinical stage (III-IV/ I-II) was performed using a Cox proportional hazards model. We compared the two TLG groups (TLG ≥ 5.4; TLG < 5.4) for the univariate disease-specific survival, locoregional recurrence-free survival, distant metastasis-free survival, and lung metastasis-free survival, which were calculated by the Kaplan–Meier method. The target events were defined as death from pharyngeal or laryngeal tumor for disease- specific survival, local or regional recurrence for locoregional recurrence-free survival, distant metastasis for distant metastasis-free survival, lung metastasis for lung metastasis-free survival, as described previously [3, 16]. Statistical significance was defined as a *P* value of less than 0.05.
